# Nature’s Anti-Fat Remedy

**Published:** 1893-12

**Authors:** 


NATURE'S ANTI-FAT REMEDY.—We do not know that the extreme heat of summer will directly cause an absorption of the anti-fat of the body, yet if there is ever an excuse for the loss of flesh it ought to be at such a time as this. It seems therefore, quite out of place to mention any kind of an anti-fat remedy other than a temperature of 100 degrees in the shade. However, if any of our readers prefer the cool breeze of the mountains, and at the same time are heavily burdened with adipose tissue, we cannot do better than recommend to them Phytoline (Walker), the anti-fat remedy of the day. We cannot give the philosophy of its action, but the clinical reports indicate that it is a drug capable of accomplishing what is claimed for it.—Extract from "Food."

